# Bacterial Cellulose Production Using the Corinthian Currant Finishing Side-Stream and Cheese Whey: Process Optimization and Textural Characterization

**DOI:** 10.3390/foods8060193

**Published:** 2019-06-04

**Authors:** Argyro Bekatorou, Iris Plioni, Konstantina Sparou, Renia Maroutsiou, Panagiota Tsafrakidou, Theano Petsi, Eleana Kordouli

**Affiliations:** 1 Department of Chemistry, University of Patras, 26500 Patras, Greece; plioni@upatras.gr (I.P.); konstantinasparou@gmail.com (K.S.); mrtsrenia@gmail.com (R.M.); thpetsi@upatras.gr (T.P.); ekordouli@upatras.gr (E.K.); 2 Department of Chemical Engineering, University of Patras, 26500 Patras, Greece; panag.tsafrak@gmail.com

**Keywords:** bacterial cellulose, Corinthian currant finishing side-stream, cheese whey, *Κοmagataeibacter sucrofermentans*, optimization, textural analysis, functional foods

## Abstract

The aim of this work was to develop bioprocesses to produce a high-value microbial product, bacterial cellulose (BC), utilizing the industrial side-stream of Corinthian currants finishing (CFS), with/without the addition of N-sources and cheese whey, and at various process conditions (temperature, pH level, and sugar concentration). For the optimization of BC production, the response surface methodology based on the central composite design was applied. Among the possible retrieved combinations, the most ideal conditions for BC in CFS extracts supplemented with N-source were 28 °C, pH 6.42, and 46.24 g/L concentration of sugars. In a similar manner, the best conditions for BC production in CFS/whey mixtures were pH 6.36, 50.4% whey percentage in the mixture, and 1.7% yeast extract. The textural characteristics of the produced BC, at different times of production and using different drying methods, were studied by scanning electron microscopy, X-ray diffractometry, porosimetry, Fourier-transform infrared spectroscopy, and thermogravimetric/differential thermal analysis, revealing increased porosity of BC compared with delignified cellulosic materials of plant origin, and a level of crystallinity that depended on the BC production time. The proposed methodology can be used to produce foods with potential prebiotic properties, using the highly nutritious CFS and the abundant cheese whey effluent as raw materials.

## 1. Introduction

The food industry plays a fundamental role in national manufacturing industries and in the economy, worldwide. However, in order to remain competitive, it should focus on openness and extroversion, product quality, and the promotion of national brands, which can be achieved by investing in specialized human resources, new technologies, and innovation [1]. Currently, white biotechnology (industrial applications of biotechnology with enzymes and micro-organisms as key tools) is the cornerstone of the future industrial economy, paving the way for energy and environmentally sustainable processes, transforming raw materials into nutritious, valuable products with minimized waste generation [2]. However, many of these efforts are not applied because of drawbacks related to productivity, ease of application and production cost. However, low-cost renewable resources still have a high potential to develop innovative and efficient biological conversion systems.

Raisins are dried grapes produced by sun, shade, or mechanical drying. In Greece, the famous Corinthian currant variety (small black *Vitis vinifera* L. raisins) is an old, historic product that played an important role in the development of the Greek State as an important exporting commodity (currently accounting for about 80% of the total global production). The Greek market absorbs less than 2% of the total production. The Corinthian currant is a dual-use variety of grapes that can be used as snack raisins or to produce wine. The current area used for the cultivation of raisins in Greece is about 10,000 ha. They are cultivated in non-irrigated, sloped mountainous areas of 300–1200 m in altitude. These areas have been reduced by about 5000 ha in the last 15 years because of the high cultivation and processing costs, as well as because of the lack of governmental subsidies. Their production and quality are also highly affected by occasional and long-term climate changes (global climate change). 

There are three main sub-varieties of the product: the *Gulf*, the *Provincial*, and the *Vostitsa* currants. *Vostitsa* is the top quality, produced exclusively in the area of Aeghion (Latitude 38°14’54’’N; Longitude 22°04’54’’E), where soil-climatic conditions, with the effects of sea breeze and sunshine, are ideal. It is the highest quality type of Corinthian currant, standing out for its unique flavor, and is exported to almost all the countries in the world, but mainly England. *Vostitsa* is a product of designated origin (PDO) (Ministerial Decree No. 442597, 1993; Commission Regulation No. 1549/98, 1998) [3,4]. 

In recent years, companies working with this product have made a lot of progress and innovation, including engagement in intense research activities to determine the nutritional value of the product. The published research shows that the *Vostitsa* currants are an excellent source of antioxidant polyphenols and retain their antioxidant activity during processing [5,6,7]. They are also rich in fiber with potential prebiotic properties, they present high bioavailability of micronutrients [8], they have anticancer properties [9], and have a moderate Glycemic Index, albeit their sweetness, therefore they can be consumed by patients with diabetes [10].

A company that processes currants generates a large amount of a lower quality side-stream (~5% of total production), with ~70% invert sugar content. In Greece, this side-stream is mainly used for vinegar production and to a lesser extent for raisin syrup production. This nutritional side-stream has a huge potential for biotechnological exploitation as substrate to produce a variety of added-value products (fermented foods, single cell proteins, and valuable microbial metabolites). The solid residues can also be valorized in a biorefinery manner through the recovery of functional food formulations (antioxidant polyphenols, prebiotic fiber, etc.). 

Bacterial cellulose (BC) is a nano-fibrous material used as an emulsifier and gel forming agent in foods, textiles, and cosmetics, as well as for medical purposes (wound dressings, burn treatments, and medical devices), and advanced material applications (biosensing materials, etc.) [11]. Its structural, physicochemical, and mechanical properties are superior to those of plant cellulose—it has high purity, increased water holding ability, strength, and better moldability. BC is produced by various species, such as certain algae and bacteria (*Acetobacter*, *Rhizobium*, *Agrobacterium*, *Salmonella*, *Escherichia*, *Sarcina*, etc.) [11]. Despite its high application potential, the BC production cost in synthetic media is prohibitive. Efforts using low cost agri-food waste substrates have shown that optimized production for industrial applications requires small-scale fermentation, selection of effective producing strains, and optimized conditions (temperature, pH level, dissolved oxygen, medium composition, agitation speed) [11]. BC can also form nanomaterials [12] and composites with other polymers (chitosan, collagen, polyaniline, silk-sericin), and nanoparticles (Ag, ZnO, TiO_2_, C-nanotubes) that have many practical applications [11]. 

The aim of this study was the development of a novel bioprocess for the production of BC utilizing the Corinthian currant finishing side-streams (CFS) as raw material, including optimization of the BC production conditions (sugar concentration of the CFS extracts, pH level, temperature, and addition of nitrogen (N)-source or cheese whey), drying of the produced BC with various techniques, and its surface and physicochemical characterization by scanning electron microscopy (SEM), X-ray diffractometry (XRD), Fourier transform-infrared spectroscopy (FT-IR), N_2_ adsorption/desorption porosimetry analysis, and thermogravimetric/differential thermal analysis (TGA/DTA). 

## 2. Materials and Methods

### 2.1. Microorganism and Media

*Κοmagataeibacter sucrofermentans* (DSM No. 15973), isolated from black cherries, was supplied by the Leibniz Institute DSMZ-German Collection of Microorganisms and Cell Cultures (Deutsche Sammlung von Mikroorganismen und Zellkulturen GmbH). This strain has been established as the model-microorganism for the study/production of BC because of its high production ability utilizing a variety of carbon and N-sources [13]. In this study, it was initially grown in petri dishes on solid medium containing (% *w*/*v*) glucose, 10; CaCO_3_, 2; yeast extract, 1; and agar, 1.5, in deionised water. The pH level was adjusted to 6.0 by the addition of 5 M NaOH solution. A stock culture was then prepared by inoculation in a Hestrin–Schramm medium, commonly known as a HS-glucose medium, consisting of (% *w*/*v*) glucose, 2.0; bacterial peptone, 0.5; yeast extract, 0.5; Na_2_HPO_4_, 0.27; and citric acid, 0.115, in deionised water. The pH level was adjusted to 6.0 by the addition of 5 M NaOH and the medium was sterilized for 15 min at 120 °C and 1–1.5 atm. Growth was carried out in 250 mL Erlenmeyer flasks containing 200 mL of the HS-glucose medium for 4 days at 30 °C [14]. 

CFS were supplied by the Agricultural Cooperatives’ Union of Aeghion S.A. (Aeghion, Greece). For BC production, the CFS were blended and then extracted with warm water at 70 °C (to avoid fermentation of the contained sugars) at about a 1:1 weight ratio, until an extract of 15 °Be (Baume hydrometer density) was obtained. The extract was further clarified by centrifugation at 6000 rpm, for 10 min and was frozen at −18 °C until the subsequent BC production experiments. 

Cheese whey was obtained from the regional dairy industry Achaia Milk Industry S.A. (AVIGAL S.A.) (Valmantoura, Achaia, Greece). It was the liquid that remained after the production of feta cheese and after the removal of whey proteins (for whey cheese production). It contained about 5% *w*/*w* lactose, 0.8% *w*/*w* proteins, and had a pH level of 6.5.

### 2.2. Optimization of BC Production in CFS Extracts

Initially, preliminary experiments to investigate the necessity of N-source addition in the CFS extracts for BC production at 30 °C were carried out. The substrates—based on the CFS extracts—were fixed so as to contain 20 g/L sugars, were supplemented with 0.5% *w*/*v* peptone or 0.5% *w*/*v* yeast extract (Table 1), and the pH level was adjusted to 3.8 or 6.8. These amounts of N-sources were similar to those contained in the synthetic HS-glucose medium. The experimental results showed that the addition of both sources (peptone and yeast extract) increased the BC yield, and therefore the subsequent experiments were carried out by adding both N-sources (at 0.5% *w*/*v* each).

For the BC production experiments, 100 mL of CFS extract, 0.5% *w*/*v* peptone, and 0.5% *w*/*v* yeast extract were mixed in 250 mL Erlenmeyer flasks. The pH of the substrates was adjusted as described below (Section Experimental Design and Statistical Analysis) and the Erlenmeyer flasks were sterilized by autoclaving for 15 min at 120 °C and at 1–1.5 atm. After this, 12 mL of sterilized substrate were transferred into Petri dishes and were inoculated with 0.5 mL of the *K. sucrofermentans* stock culture. The Petri dishes were incubated for BC production for 7 days at different conditions as described below (Section Experimental Design and Statistical Analysis). 

To purify the produced hydrated BCs from any medium residues and bacterial cells, they were immersed into 5 M NaOH solution for 1 h at 90 °C [14], and were then rinsed several times with sterile deionised water until the pH level became neutral. The final clarified products were oven-dried at 70 °C overnight and weighed for yield estimation, or were freeze-dried by cooling to −40 °C and with an overnight exposure to vacuum (5–15 × 10^−3^ bar at −45 °C) on a freeze dry system, Freezone 4.5 (Labconco Corp., Kansas, MO, USA). The dried BCs were used for further physicochemical/textural analysis.

#### Experimental Design and Statistical Analysis

Response surface methodology (RSM) is useful in studying and optimizing multivariate processes, combining mathematical and statistical tools to evaluate the effects of several factors, with a small number of experiments required to investigate their possible interactions. The central composite design (CCD) is a widely used RSM when upper and lower limits of each factor are set for an experimental design [15]. The significant interactions between the variables and the combination of factors generating a certain optimal response can be identified. The optimization of BC production using the RSM/CCD methodology was based on the correlation of BC production yield with the levels of a number of variables that potentially affect it, in a particular area of interest. Specifically, a 2^3^ full factorial design was used with six repetitions at the central point. A total of 20 runs were performed, in which three independent variables (sugar concentration in the substrate, substrate pH level, and process temperature) were studied (optimization *Experiment 1*). The experiment was designed using the Design-Expert version 10 Software (Stat-Ease, Inc., Minneapolis, MN, USA). The independent variables were coded as X_1_, X_2_, and X_3_, and had three levels: −1, 0, +1. The encoded value 0 corresponds to the center point, while the −1 and +1 correspond to the lower and upper values of each variable. The dependent variable was the yield of BC production. The combinations of the independent variables used in this experimental design are shown in Table 2. The experiments were repeated in triplicate and the average values are presented.

In a similar manner, a second optimization experiment was carried out (*Experiment 2*) with a total of 20 runs, in which the three independent variables were the concentration of cheese whey in a mixture of cheese whey and CFS extract, the substrate pH level, and the addition of N-source (yeast extract) (Table 2). The BC production temperature in this case was held constant at 30 °C.

In both cases, in order to evaluate the model, the coefficient of multiple regression R^2^ was calculated, showing the model’s goodness-of-fit in the data, and the coefficient F of the corresponding ANOVA test, which indicates how far the data are scattered from the mean. After selecting the best predicting model, the best combination of the independent variables that leads to maximizing the response was calculated.

Finally, an experiment was performed using the optimum predicted combination of values of the independent variables, and the obtained value was compared with the predicted value for BC yield.

### 2.3. Analytical Methods 

#### 2.3.1. Determination of Sugars 

Sugars were analyzed on a Shimadzu LC-9A HPLC system consisting of a Nucleogel Ion 300 OA column, a LC-9A pump, a RID-6A refractive index detector, a CTO-10A column oven, and a DGU-2A degassing unit. An aqueous solution of 0.008 M H_2_SO_4_ was used as the mobile phase (flow rate 0.5 mL/min). The column temperature was 33 °C. The sample dilution was 0.4% *v/v* and the injection volume was 40 μL. Ultra-pure water obtained by a Milli-Q water purifying system (resistivity 18.2 MΩ cm^−1^) was used for the dilutions. Samples were filtered through 0.2 μm microfilters before injection. The sugar (g/L) concentrations were calculated using standard curves.

#### 2.3.2. Scanning Electron Microscopy (SEM)

The morphology of the dried BC samples (produced as described in Section 2.2) was observed by field emission SEM on a FE-SEM, FEI InspectTM F50 instrument based at the Technological-Educational Institute of Western Greece (Patras, Greece).

#### 2.3.3. Textural Characteristics Determined by N_2_ Adsorption/Desorption Experiments 

The textural characteristics of the BC samples were carried out at 77 K using a Micromeritics apparatus (Tristar 3000 porosimeter). The specific surface area, the mean pore diameter, the specific pore volume, and the pore size distribution of the produced BCs were determined by measuring the amount of N_2_ adsorbed on and desorbed from the material surface over a wide range of relative pressures. Dried samples (0.4–0.6 g) were degassed using N_2_ flow at 110 °C for 90 min in order to remove any gases adsorbed in the samples’ surface. The specific surface area was calculated by the Brunauer–Emmett–Teller (BET) equation. Pore size distribution of the samples was estimated by the Barrett–Joyner–Halenda (BJH) method using the desorption data [16].

#### 2.3.4. Thermogravimetric/Differential Thermal Analysis (TGA/DTA)

The TGA/DTA analysis of the BC samples was carried out in a Perkin Elmer Diamond TGA/DTA system under N_2_ atmosphere (flow 200 mL/min). For moisture removal, the samples were placed in an aluminum crucible and heated at 100 °C until their weight was stabilized. Afterwards, the temperature was increased from up to 600 °C (rate 10 °C/min) and the weight loss graphs were obtained.

#### 2.3.5. Fourier Transform-Infrared Spectroscopy (FT-IR) Analysis

For FT-IR analysis, about 2 mg of each sample was mixed with 200 mg KBr (spectroscopic grade) and the mixture was pressed in a hydraulic press (8 ton) for 5 min. Thereafter, the IR spectra in the range 4000–400 cm^−1^ were recorded on a Perkin–Elmer spectrometer (Waltham, MA, USA) with a resolution of 4 cm^−1^. Each sample was scanned 10 times.

#### 2.3.6. X-ray Diffraction Analysis (XRD)

To determine the crystalline nature of BC, the XRD patterns were obtained using a Bruker (Billerica, MA, USA) D8 Advance apparatus equipped with CuKa radiation source that was Ni-filtered. The intensity of diffracted radiation was measured between 5–60° (2*θ*) with a scanning rate of 0.1 °/min. The crystallinity index (C_I_) was calculated using the Segal equation [17]:

(1) $$C_{I}\left(\%\right)=\left(\frac{I_{max}-I_{min}}{I_{max}}\right)\times 100$$

where I_max_ is the intensity of the strongest XRD peak at 2*θ* = 22° and I_min_ is the intensity of the background at 2*θ* = 18°.

The mean size of cellulose crystallites was calculated by the Scherrer’s equation [18]:

(2) $$\beta =\frac{Κ\lambda }{L\;cos\theta }$$

where *β* is the width at half maximum of a selected peak, K is a constant (0.94), λ is the wavelength of the Cu Kα X-ray radiation (λ = 0.15418 nm), *θ* is the diffraction angle, and L is the mean crystallite length.

## 3. Results and Discussion

### 3.1. Optimization of BC Production in CFS Extracts by RSM/CCD

For optimization of the BC production in the CFS extracts, a set of preliminary experiments were carried out with and without the addition of a N-source (peptone or yeast extract) to evaluate the need of N-supplementation. The results are shown in Table 1. It can be observed that, as expected, the N-source addition had a positive effect, leading to a significant boost in the final BC production yield. Specifically, at both the tested pH values, the BC yield with the addition of peptone and yeast extract was more than doubled. Based on these experimental results, subsequent BC production optimization experiments were carried out by adding both N-sources (at 0.5% *w*/*v* each) in the substrates. 

In the second set of experiments (optimization *Experiment 1*), for the production of BC in N-supplemented CFS extracts using the RSM/CCD methodology (actual and coded values shown in Table 2), a large similarity between the experimental and the predicted values was observed after the mathematical processing, which implies that the model has great credibility (Table 3). The second-order linear regression equation was obtained, which describes the relation between the dependent variable and the independent variables:

BC (g/L) = −33.08 + 0.48X_1_ + 8.09X_2_ + 0.92X_3_ +0.0015X_1_X_2_ + 0.0003X_1_X_3_ + 0.048X_2_X_3_ − 0.0055X_1_^2^ − 0.76X_2_^2^ − 0.02X_3_^2^. (3)

The positive coefficients for the model (X_1_, X_2_, Χ_3_, X_1_X_2_, X_1_X_3_, X_2_X_3_) mean that they act synergistically on BC production, while the negative factors (X_1_^2^, X_2_^2^, Χ_3_^2^) mean they have an adverse effect. The model’s statistical significance was determined by the *F*-test for the analysis of variance (ANOVA) (Table 4), where the regression was shown to be significant at the 5% significance level (*p* < 0.05). The probability *p* (<0.0001) was very small, showing the importance of the model. The *F* value of the model (69.43) implies that the model is statistically significant with a high degree of confidence. In addition, the calculation of the variance coefficient (CV) describes the extent of the data dispersion and expresses the precision and repeatability of this assay. Small CV values show a good degree of repeatability. In this case, the CV was 4.47%, which is very satisfactory. The high R^2^ value (0.98), which expresses the percentage of variability of the dependent variable interpreted by the independent variables, shows that 98% of the variability of BC production is interpreted by the independent variables studied. The predicted *R*^2^ of 0.89 is in reasonable agreement with the *R_Adj_*^2^ value (0.97), which shows the reliability of the model (Table 4).

**Figure 1:**
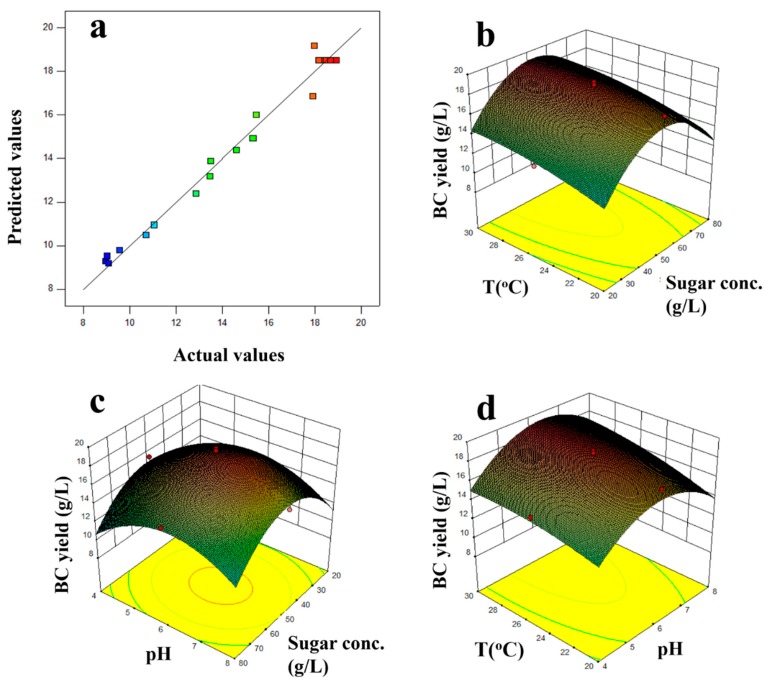
(**a**) Predicted values against experimental data of bacterial cellulose (BC) yield (g/L) according to the experimental design, and three-dimensional (3D)-imaging of the BC yield response surface at (**b**) varying pH levels and sugar concentration (Sugar conc.), (**c**) varying sugar concentration and temperature (T), and (**d**) varying pH levels and temperature (*Experiment 1*).

Figure 1a illustrates the correlation of the predicted values with the experimental data. The normal distribution of the data indicates that the model is reliable for predicting the production of BC. Analyzing the three-dimensional (3D) representation of the response surface, as shown in Figure 1b–d, each time keeping one of the three evaluated parameters constant provides useful information on the relationship between the studied parameters and the dependent variable.

The software indicated a total of 10 possible combinations of factor values for optimization of the result (Table 5). Among the possible combinations, the most ideal was solution no. 3—the temperature was approximately 28 °C (28.07 °C), which is the optimal growth temperature of the specific bacteria, the pH level of the substrate was 6.42, which means that it needed minimal further adjustment after preparation (the pH value of the substrate was ~6.2 after sterilization), and most importantly, the concentration of sugars in the substrate was the lowest compared with all possible combinations (46.24 g/L), which means that it is possible to produce high concentrations of BC with a low concentration of fermentable sugar in the substrate. 

The predicted value of the BC yield using the above optimal combination of factors in the mathematical model was 18.95 g/L. The confirmation of the value was done by determining the experimental value of the BC yield after repeating the experiment with the best obtained factor values. Specifically, three experiments were performed under these optimal conditions and the average of the BC yield obtained was 18.4 ± 0.6 g/L.

### 3.2. Optimization of BC Production in CFS Extracts and Cheese Whey by RSM/CCD

In another set of experiments (optimization *Experiment 2*), for the production of BC in CFS extracts supplemented with cheese whey or N-source (yeast extract), at a constant temperature (30 °C) and at different substrate pH values (Table 2), using the RSM/CCD methodology, similar results were obtained regarding the credibility of the model. In brief, the second-order linear regression equation that was obtained is shown in Equation (4):

BC (g/L) = −23.84 + 12.56X_1_ + 0.2X_2_ + 5.76X_3_ − 1.03Χ_1_^2^ − 0.002Χ_2_^2^ − 6.42Χ_3_^2^ − 0.021X_1_X_2_ + 0.631X_1_X_3_ + 0.005X_2_X_3_. (4)

The model’s statistical significance was determined by ANOVA analysis, where the regression was shown to be significant at the 5% significance level (*p* < 0.05), the probability *p* (<0.0001) was very small, the CV was 4.47%, the *R*^2^ value was 0.97, and the *R_Adj_*^2^ value was 0.88, which shows the reliability of the model. As in the case of the optimization *Experiment 1*, the software indicated 10 possible combinations of factor values for the optimization of the result. The most ideal solution was the combination of substrate pH 6.36, 50.4% whey percentage in the substrate, and the addition of 1.7% yeast extract. The predicted value for BC yield using the above optimal combination of factors in the mathematical model was 19.22 g/L. The confirmation of the value was done by determining the experimental value of the BC yield after repeating the experiment with the best model factor values. Specifically, three experiments were performed under these optimal conditions and the average yield of BC obtained was 18.9 ± 0.7 g/L. These results indicate that cheese whey, which is a cheaper and abundant food industry effluent, can be used at ratios up to 1:1 with the rarer and more expensive CFS extracts as substrate to produce BC. However, in this case, the addition of a more expensive N-source is also required.

### 3.3. Different Processing Techniques for the Produced BC

**Figure 2:**
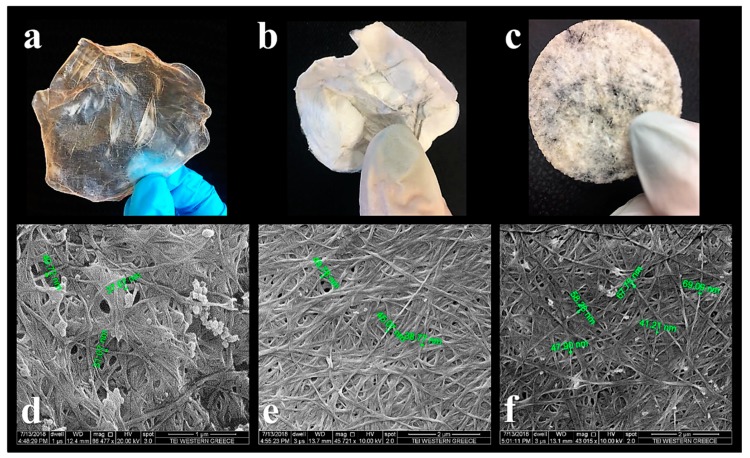
Different bacterial cellulose (BC) textures: (**a**) oven-dried BC, (**b**) freeze-dried BC, (**c**) wet-grinded and then freeze-dried BC, and their corresponding scanning electron microscopy (SEM) images [(**d**): ×86,477, (**e**): ×45,721, and (**f**): ×43,015] (*Experiment 1*).

As shown in Figure 2, the produced BC was processed (dried) by different methods, leading to different forms and textures. Figure 2a shows the oven-dried BC having a clear polymeric film-like appearance and texture. Figure 2b shows the freeze-dried BC sample having a completely different look, resembling paper or cotton fabric, without transparency, but with similar ductility. When wet BC was grinded and then freeze-dried, a sponge-like product was obtained (Figure 2c). As it can be seen in the SEM images of Figure 2d–f, the produced BC consisted of entangled—but with a relatively high degree of orientation—fibers that create a dense 3D network. The smaller sized BC fibers had widths around 30–70 nm as determined by SEM.

### 3.4. Textural Characteristics of the BC Samples 

Table 6 presents the textural characteristics of the BC samples as determined by N_2_ sorption/desorption measurements. The surface areas of the BC_7_ sample (BC produced in CFS extracts for 7 days) and the BC_HS_ sample (produced in HS medium) was identical (6.5 m^2^/g), whereas the BC_4_ sample (produced in CFS extracts for 4 days) had a smaller surface area (4.2 m^2^/g). Similar results were obtained for the volume and the pore diameters of the BC samples.

Compared with samples from different types of delignified cellulosic biomasses (mango wood 1.1 m^2^/g; sal wood 0.6 m^2^/g; rice husk 6.6 m^2^/g; all of Indian origin), previously analyzed using the same N_2_ sorption/desorption technique [19], as well as softwood sawdust (0.85 m^2^/g) [20], it can be observed that BC has a larger cumulative surface area than the wood cellulosics, but comparable with that of rice husk. Therefore, BC compared with delignified wood cellulose is a more porous material. Moreover, it consists of pure cellulose, thus it does not require chemical pretreatment (e.g., delignification), which is usually applied to wood biomasses in order to remove non-cellulosic components and increase their porosity for various applications [20]. The average pore diameter of the BCs (>176 Å) was higher than those of the delignified wood samples and rice husk (80–174 Å) [19,20].

**Figure 3:**
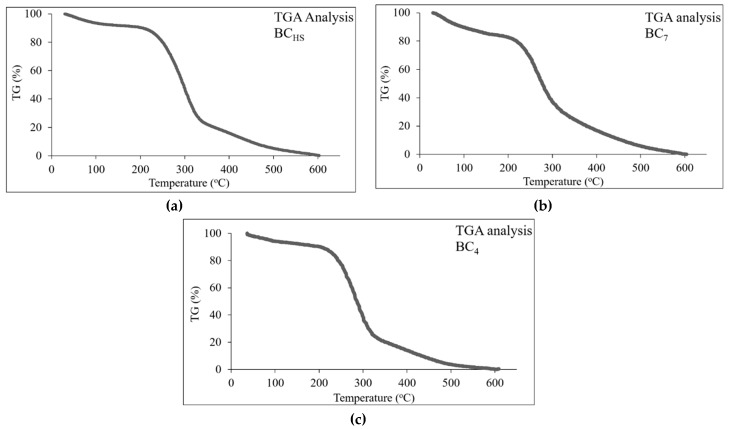
Weight loss diagram during the thermogravimetric/differential thermal analysis (TGA analysis) of the bacterial cellulose (BC) samples: (**a**) BC_HS_ [produced in Hestrin–Schramm (HS) medium for 7 days], (**b**) BC_4_ and (**c**) BC_7_ (produced in the Corinthian currant finishing side-stream extracts, supplemented with N-source for 4 and 7 days, respectively).

Figure 3 shows the % weight loss of the samples during the TGA/DTA analysis. All samples exhibited similar behavior. Specifically, sample BC_7_ (Figure 3c) presented a smoother weight loss rate than the other two samples that behaved almost identically (Figure 3a,b). The BC fibers in this sample, because of their longer developing process in the nutrient substrate, seem to be more strongly bound, thus resisting thermal decomposition. The initial decomposition interval at about 150 °C was common for all samples, and weight loss at this stage was probably due to moisture removal. The highest weight loss rate occurred in all three samples up to 300 °C. The maximum % weight loss occurred at 220–400 °C as expected because of cellulose thermolysis (~350 °C). After 450 °C, another decaying stage can be distinguished because of further pyrolysis of the samples.

**Figure 4:**
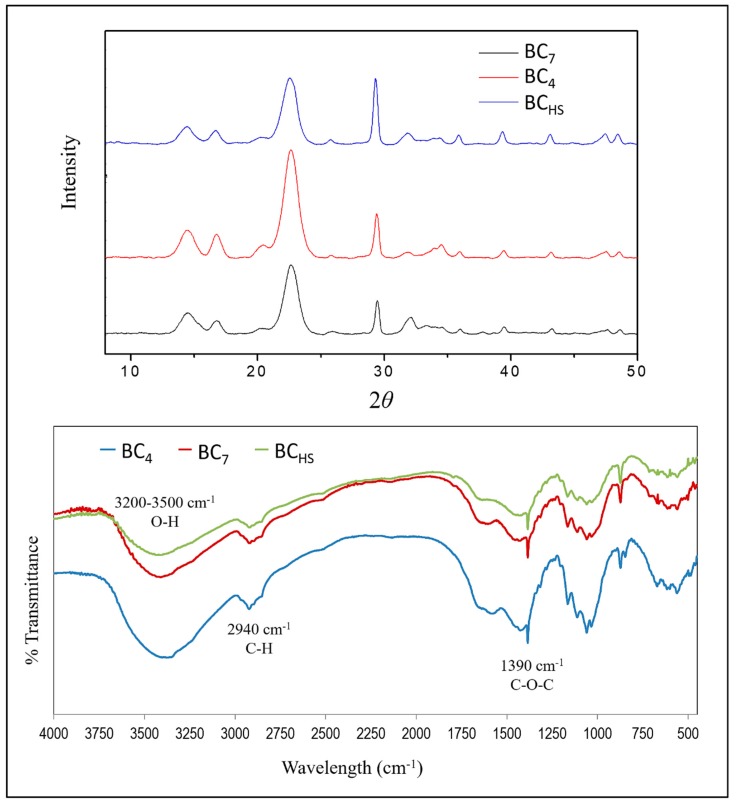
X-ray diffraction spectra (**Up**), and Fourier-transform infrared spectra (**Down**) of the bacterial cellulose (BC) samples: (a) BC_HS_ [produced in Hestrin–Schramm (HS) medium for 7 days], (b) BC_4_ and (c) BC_7_ (produced in the Corinthian currant finishing side-stream extracts, supplemented with N-source for 4 and 7 days, respectively).

The FT-IR analysis provided the molecular vibration spectra of the BC samples due to IR absorption. Variability of the IR absorption intensity indicated structural changes in the samples. Similar absorptions for all samples were observed and no substantial differences could be identified (Figure 4). In particular, absorptions with characteristic peaks at 3300, 2940, and 1390 cm^−1^ were attributed to the cellulosic bond vibrations of O–H, C–H, and C–O–C, respectively, usually obtained from BC [21]. Peak absorption at 1390 cm^−1^ was attributed to the presence of carbonyl groups in BC [22]. The peaks at 3490 and 2940 cm^−1^ are typical for O–H and C–H bond vibrations [23] with the latter being attributed to aliphatic compounds. The absorption range corresponding to the hydroxyl group and the hydrogen bond was observed at 3200–3500 cm^−1^, which is consistent with the literature [24].

XRD spectroscopy, on the other hand, provides information on the crystalline structure of the studied solid materials. In Figure 4, three wide peaks at 2*θ* of 14.7°, 16.8°, and 22.8° were observed for all BC samples. These peaks were attributed to the cellulose structures Iα and Iβ. At 14.7° the (1 0 0), (1 1 0); at 16.8° the (0 1 0), (1 1 0); and at 22.8° the (1 1 0), (2 2 0) levels of BC Iα and Iβ were observed, respectively. The range of the three characteristic peaks was due to the partial crystallinity of BC cultured under static conditions, with the peak at 16.8° being the most characteristic for bacteria-produced cellulose structure Iα. As shown in Table 6, the BC_7_ crystallinity index was similar to BC_HS_, whereas BC_4_ was lower. This was probably due to the shorter stay of BC_4_ in the nutrient substrate, providing less time for further crystallization, which led to more amorphous areas in the BC_4_ structure.

## 4. Conclusions and Future Work

CFS is a material of low cost but with a similar chemical composition and nutritional value as the originating product (Corinthian currants). This work showed that CFS can be used as substrate for the production of added-value (bio)products. To fully exploit this highly nutritional side-stream, the contained sugar was converted to BC with a good yield during a 7-day process. In a parallel study, the CFS extract was used for the production of good quality white wine using immobilized yeast, while the solid residue was extracted using different solvents to recover antioxidant phenolics in a biorefinery manner, showing that hydroalcoholic extraction or ethanol/methanol combinations are the best treatments for this purpose [25].

The BC produced in this work and all substrates were food grade. Therefore, this methodology can be used to produce foods with prebiotic properties, for example similar to the traditional Asian food nata-de-coco, using the highly nutritious and delicious CFS and the abundant cheese whey effluent as raw materials. Whey can also be evaluated after various proteolytic pretreatments as a sole N-source, which is the aim of ongoing studies. 

The findings of the textural analysis carried out in this work are also useful in the case that BC will be used for other food or non-food purposes (e.g., fabrication of BC-based biocomposites for antibacterial films [26], thermoplastic polymers, and other materials [27]). 

Finally, the exploitation of CFS in a biorefinery manner for food products (prebiotic/antioxidant foods), biobased materials (BC), and food additives (antioxidant phenolics), will maximize the profits of the raisin processing companies, that will also be able to subsidize the grape farmers who gradually abandon the cultivation of raisins because of intolerable production costs and lack of governmental subsidies. An environmental impact is also expected because of waste minimization. 

## Figures and Tables

**Table 1:** Bacterial cellulose (BC) production in Corinthian currant finishing side-stream extracts (20 g/L sugar concentration), at different pH values, and with/without the addition of N-sources (peptone and yeast extract) (7-day process).

pH	N-Source	BC Yield	
		(g/L)	(g/g Sugar)
3.8	None	7.37	0.04
3.8	Peptone	11.78	0.06
3.8	Yeast extract	18.26	0.09
6.8	None	8.65	0.04
6.8	Peptone	18.91	0.09
6.8	Yeast extract	16.46	0.08

**Table 2:** Actual and coded factor values of the response surface methodology for optimization of bacterial cellulose (BC) production in the Corinthian currant finishing side-stream (CFS) extracts.

Independent Variable	Unit	Symbol	Coded Values		
			−1	0	1
*Experiment 1*: Addition of N-source					
Sugars concentration in the CFS substrate	g/L	X_1_	20	50	80
pH		X_2_	4	6	8
Temperature	°C	X_3_	20	25	30
*Experiment 2:* Addition of cheese whey					
pH		X_1_	3.8	6	8.2
Whey percentage in the CFS substrate	% *v/v*	X_2_	0	35	70
Yeast extract addition	% *w*/*v*	X_3_	0	0.5	1

**Table 3:** Experimental design and results of the response surface methodology for optimization of bacterial cellulose (BC) production in the Corinthian currant finishing side-stream extracts (*Experiment 1*; average values of three repetitions).

Run	Coded Values			BC Yield (g/L)	
	X_1_	X_2_	X_3_	Experimental Value	Predicted Value
1	−1	−1	−1	8.99	9.28
2	1	−1	−1	9.03	9.74
3	−1	1	−1	9.09	9.11
4	1	1	−1	9.59	9.93
5	−1	−1	1	10.71	10.46
6	1	−1	1	11.07	11.13
7	−1	1	1	12.88	12.22
8	1	1	1	13.47	13.25
9	−1	0	0	13.51	13.81
10	1	0	0	14.63	14.55
11	0	−1	0	15.33	15.02
12	0	1	0	15.47	15.99
13	0	0	−1	17.90	16.92
14	0	0	1	17.98	19.17
15	0	0	0	18.43	18.55
16	0	0	0	18.53	18.55
17	0	0	0	18.93	18.55
18	0	0	0	18.47	18.55
19	0	0	0	18.17	18.55
20	0	0	0	18.68	18.55

**Table 4:** ANOVA for quadratic model of the response surface methodology for optimization of bacterial cellulose production in the Corinthian currant finishing side-stream extracts (*Experiment 1*).

Source	Sum of Squares	Degrees of Freedom	Mean Square	*F*-Value	Probability (*P*) > *F*
Model	263.53	9	29.28	69.43	< 0.0001
Residual	4.22	10	0.42		
Lack of fit	3.89	5	0.78	11.95	0.0083
Pure error	0.33	5	0.065		
Total	267.74	19			

*R*^2^ = 0.98, variance coefficient (CV) = 4.47%, *R_Adj_*^2^ = 0.97, predicted *R*^2^ = 0.88.

**Table 5:** Possible combinations of the three optimization factors (independent variables) of the response surface methodology for optimization of bacterial cellulose (BC) production in the Corinthian currant finishing side-stream extracts (*Experiment 1*).

Combination	Sugar Concentration (g/L)	pH	Temperature (°C)	BC Yield (g/L)
1	56.13	6.32	28.89	19.04
2	51.84	6.10	27.79	19.02
3	46.24	6.42	28.07	18.95
4	53.83	6.06	28.52	19.05
5	53.34	6.53	28.26	19.03
6	49.88	6.27	28.90	19.16
7	51.12	6.18	28.87	19.15
8	50.38	6.73	28.22	18.94
9	47.43	6.34	28.06	19.01
10	48.88	6.07	27.89	19.00

**Table 6:** Textural characteristics of bacterial cellulose (BC) samples produced in the Corinthian currant finishing side-stream extracts (CFS) supplemented with nitrogen (N)-source or cheese whey.

Parameter	BC Sample					
	BC_HS_		BC_4_		BC_7_	
	N-Source	Whey	N-Source	Whey	N-Source	Whey
Surface area (m^2^/g)	6.5	6.0	4.2	5.0	6.5	8.0
Average pore diameter (Å)	201	204	176	195	220	229
Cumulative pore volume (cm^3^/g)	0.04	0.04	0.03	0.06	0.05	0.04
Crystallinity Index (%)	70.6	72.4	64.6	64.4	70.0	70.1
Crystallite size (Å)	32.4	31.9	29.4	28.4	34.0	31.8

BC_HS_, produced in Hestrin–Schramm (HS) medium for 7 days; BC_4_, produced in CFS extract for 4 days; BC_7_, produced in CFS extract for 7 days.
